# Supplementary material for: The history of mesowear: a review
Source: PeerJ. 2020 Feb 13;8:e8519. doi: 10.7717/peerj.8519 (PMC7024573; doi:10.7717/peerj.8519)
Supplement: Supplemental Information 2 [file peerj-08-8519-s002.docx]

**Annex 1: Raw data references**

**The history of mesowear: A review**

Nicole L. Ackermans^a^

^a^ Clinic for Zoo Animals, Exotic Pets and Wildlife, Vetsuisse Faculty, University of Zurich, Zurich, Switzerland.

[nlackermans@gmail.com](mailto:Nicole.ackermans@uzh.ch)

**References**

**Ackermans, N. L., Martin, L. F., Codron, D., Hummel, J., Kircher, P. R., Richter, H., Clauss, M. and Hatt, J.-M.** (in prep.). Mesowear resembles a lifetime signal in a long-term feeding experiment on sheep (*Ovis aries*). *Palaeogeography, Palaeoclimatology, Palaeoecology*.

**Ackermans, N. L., Winkler, D. E., Schulz-Kornas, E., Kaiser, T. M., Müller, D. W. H., Kircher, P. R., Hummel, J., Clauss, M. and Hatt, J.-M.** (2018). Controlled feeding experiments with diets of different abrasiveness reveal slow development of mesowear signal in goats (*Capra aegagrus hircus*). *Journal of Experimental Biology* **221**, jeb186411.

**Aiglstorfer, M. and Semprebon, G. M.** (2019). Hungry for fruit?–A case study on the ecology of middle Miocene *Moschidae* (Mammalia, Ruminantia). *Geodiversitas* **41**, 385-399.

**Altamura, S.** (2016). Dietary behaviour of the pleistocene Cretan dwarf deer:

preliminary clues from mesowear analysis of

*candiacervus ex gr. candiacervus* *ropalophorus* from Bate cave *Alpine and Mediterranean Quaternary* **29**, 77-89.

**Amano, N., Rivals, F., Moigne, A.-M., Ingicco, T., Sémah, F. and Simanjuntak, T.** (2016). Paleoenvironment in East Java during the last 25,000 years as inferred from bovid and cervid dental wear analyses. *Journal of Archaeological Science: Reports* **10**, 155-165.

**Aranguren, B., Grimaldi, S., Benvenuti, M., Capalbo, C., Cavanna, F., Cavulli, F., Ciani, F., Comencini, G., Giuliani, C. and Grandinetti, G.** (2019). Poggetti Vecchi (Tuscany, Italy): A late Middle Pleistocene case of human–elephant interaction. *Journal of Human Evolution* **133**, 32-60.

**Athanassiou, A., Roussiakis, S. J., Giaourtsakis, G. E. T., Theodorou, G. E. and Iliopoulos, G.** (2014). A new hornless rhinoceros of the genus *Acerorhinus* (Perissodactyla: Rhinocerotidae) from the Upper Miocene of Kerassiá (Euboea, Greece), with a revision of related forms. *Palaeontographica Abteilung A* **303**, 23-59.

**Barrón-Ortiz, C. I., Jass, C. N., Barrón-Corvera, R., Austen, J. and Theodor, J. M.** (2019). Enamel hypoplasia and dental wear of North American late Pleistocene horses and bison: an assessment of nutritionally based extinction models. *Paleobiology* **45**, 484-515.

**Barrón-Ortiz, C. R., Theodor, J. M. and Arroyo-Cabrales, J.** (2014). Dietary resource partitioning in the Late Pleistocene horses from Cedral, north-central Mexico: evidence from the study of dental wear. *Revista Mexicana de Ciencias Geológicas* **31**, 260-269.

**Barrón Ortiz, C. R. and Guzmán Gutiérrez, J. R.** (2009). Hábitos alimenticios de los caballos *Dinohippus mexicanus* y *Neohipparion eurystyle* del Hemphiliano Tardío de Tecolotlán, Jalisco, México. *Investigación y Ciencia* **17**, 24-29.

**Belmaker, M. and O'Brien, H. D.** (2018). Mesowear study of ungulates from the early Pleistocene site of ‘Ubeidiya (Israel) and the implications for early Homo dispersal from Africa. *Quaternary International* **480**, 66-77.

**Berlioz, E., Azorit, C., Blondel, C., Ruiz, M. S. T. and Merceron, G.** (2017). Deer in an arid habitat: dental microwear textures track feeding adaptability. *Hystrix, the Italian Journal of Mammalogy* **28**, 222-230.

**Bernor, R. L., Armour-Chelu, M., Kaiser, T. M. and Scott, R. S.** (2003). An evaluation of the Late MN9 (Late Miocene, Vallesian age), *Hipparion* assemblage from Rudabánya (Hungary): systematic background, functional anatomy and paleoecology. *Coloquios de Paleontologia* **Ext 1**, 35-46.

**Bernor, R. L., Ataabadi, M. M., Meshida, K. and Wolf, D.** (2016). The Maragheh hipparions, late Miocene of Azarbaijan, Iran. *Palaeobiodiversity and Palaeoenvironments* **96**, 453-488.

**Bernor, R. L., Gilbert, H., Semprebon, G. M., Simpson, S. and Semaw, S.** (2013). *Eurygnathohippus woldegabrieli, sp. nov.*(Perissodactyla, Mammalia), from the middle Pliocene of Aramis, Ethiopia. *Journal of Vertebrate Paleontology* **33**, 1472-1485.

**Bernor, R. L., Göhlich, U. B., Harzhauser, M. and Semprebon, G. M.** (2017). The Pannonian C hipparions from the Vienna Basin. *Palaeogeography, Palaeoclimatology, Palaeoecology* **476**, 28-41.

**Bernor, R. L. and Kaiser, T. M.** (2006). Systematics and paleoecology of the earliest Pliocene equid, *Eurygnathohippus hooijeri n. sp.* from Langebaanweg, South Africa. *Mitteilungen aus dem Hamburgischen zoologischen Museum und Institut* **103**, 149-186.

**Bernor, R. L., Kaiser, T. M. and Nelson, S. V.** (2004). The Oldest Ethiopian Hipparion (Equinae, Perissodactyl from Chorora: Systematics, Paleodiet and Paleoclimate. *Courier forschungsinstitut Senckenberg* **246**, 213-226.

**Bernor, R. L., Kaiser, T. M., Nelson, S. V. and Rook, L.** (2011). Systematics and Paleobiology of *Hippotherium malpassii n. sp.*(Equidae, Mammalia) from the latest Miocene of Baccinello V3 (Tuscany, Italy). *Bollettino della Società Paleontologica Italiana* **50**, 175-208.

**Bernor, R. L., Kaiser, T. M. and Wolf, D.** (2008). Revisiting As Sahabi equid species diversity, biogeographic patterns, and dietary preferences. *Garyounis Scientific Bulletin*, 159-167.

**Bernor, R. L., Semprebon, G. M. and Damuth, J.** (2014). Maragheh ungulate mesowear: interpreting paleodiet and paleoecology from a diverse fauna with restricted sample sizes. *Annales Zoologici Fennici* **51**, 201-209.

**Bibi, F.** (2007). Origin, paleoecology, and paleobiogeography of early Bovini. *Palaeogeography, Palaeoclimatology, Palaeoecology* **248**, 60-72.

**Blondel, C., Merceron, G., Andossa, L., Taisso, M. H., Vignaud, P. and Brunet, M.** (2010). Dental mesowear analysis of the late Miocene Bovidae from Toros-Menalla (Chad) and early hominid habitats in Central Africa. *Palaeogeography, Palaeoclimatology, Palaeoecology* **292**, 184-191.

**Blondel, C., Rowan, J., Merceron, G., Bibi, F., Negash, E., Barr, W. A. and Boisserie, J.-R.** (2018). Feeding ecology of Tragelaphini (Bovidae) from the Shungura Formation, Omo Valley, Ethiopia: Contribution of dental wear analyses. *Palaeogeography, Palaeoclimatology, Palaeoecology* **496**, 103-120.

**Boardman, G. S.** (2013). Paleoecology of Nebraska’s ungulates during the Eocene-Oligocene climate transition. In *Department of Earth and Atmospheric Sciences*, vol. PhD, pp. 134: University of Nebraska-Lincoln.

**Bravo-Cuevas, V. M. and Jiménez-Hidalgo, E.** (2015). First reported occurrence of *Palaeolama mirifica* (Camelidae, Lamini) from the Late Pleistocene (Rancholabrean) of Puebla, central Mexico. *Boletín de la Sociedad Geológica Mexicana* **67**, 13-20.

**Bravo-Cuevas, V. M., Jiménez-Hidalgo, E., Cuevas-Ruiz, G. E. and Cabral-Perdomo, M. A.** (2012). A small camelid *Hemiauchenia* from the Late Pleistocene of Hidalgo, central Mexico. *Acta Palaeontologica Polonica* **57**, 497-509.

**Bravo-Cuevas, V. M., Jiménez-Hidalgo, E. and Priego-Vargas, J.** (2011). Taxonomía y hábito alimentario de *Equus conversidens* (Perissodactyla, Equidae) del Pleistoceno Tardío (Rancholabreano) de Hidalgo, centro de México. *Revista mexicana de ciencias geológicas* **28**, 65-82.

**Bravo-Cuevas, V. M. and Priego-Vargas, J.** (2009). Dietary evaluation of a hipparionin horse population from the middle Miocene of Oaxaca, southeastern Mexico. *Revista mexicana de ciencias geológicas* **26**, 356-366.

**Bravo-Cuevas, V. M., Priego-Vargas, J., Jiménez-Hidalgo, E., Barrón-Ortiz, C. R., Theodor, J. M. and Castillo-Cerón, J. M.** (2015). Feeding ecology of *Astrohippus stockii* from the late Hemphillian of central Mexico: stable carbon isotopes analysis and dental wear patterns. *Ameghiniana* **52**, 502-517.

**Brent Jones, D. and Desantis, L. R. G.** (2017). Dietary ecology of ungulates from the La Brea tar pits in southern California: A multi-proxy approach. *Palaeogeography, Palaeoclimatology, Palaeoecology* **466**, 110-127.

**Brink, J. and Stynder, D.** (2009). Morphological and trophic distinction in the dentitions of two early alcelaphine bovids from Langebaanweg (genus Damalacra). *Palaeontologia africana* **44**, 139-193.

**Brophy, J. K., de Ruiter, D. J., Fortelius, M., Bamford, M. and Berger, L. R.** (2016). Pleistocene Bovidae (Mammalia) from Malapa, Gauteng Province, South Africa. *Palaeontologia Electronica* **19**, 1-22.

**Butler, K., Louys, J. and Travouillon, K.** (2014). Extending dental mesowear analyses to Australian marsupials, with applications to six Plio-Pleistocene kangaroos from southeast Queensland. *Palaeogeography, Palaeoclimatology, Palaeoecology* **408**, 11-25.

**Byerly, R. M.** (2007). Palaeopathology in late Pleistocene and early Holocene Central Plains bison: dental enamel hypoplasia, fluoride toxicosis and the archaeological record. *Journal of Archaeological Science* **34**, 1847-1858.

**Cammidge, T., Kooyman, B. and Theodor, J. M.** (2019). Diet reconstructions for end-Pleistocene *Mammut americanum* and *Mammuthus* based on comparative analysis of mesowear, microwear, and dental calculus in modern *Loxodonta africana*. *Palaeogeography, Palaeoclimatology, Palaeoecology*, 109403.

**Cammidge, T. S.** (2017). Comparison of Paleodietary Reconstructions Using Pre-and Post-Glacial *Mammut* and *Mammuthus*. In *Biological Sciences*, vol. PhD. Calgary, Alberta: University of Calgary.

**Capalbo, C.** (2018). Paleoecological insight into the straight-tusked elephant population from the late Middle Pleistocene site of Poggetti Vecchi, vol. PhD. Palermo: University of Palermo.

**Carbot-Chanona, G., Lagunas-Rodríguez, Z., Suárez, S. and Jiménez-Moreno, F. J.** (2017). Aspectos paleobiológicos de dos ejemplares de *Mammuthus columbi* (Mammalia, Proboscidea, Elephantidae) del Pleistoceno de Puebla, centro de México. *Boletín de la Sociedad Geológica Mexicana* **69**, 591-609.

**Clauss, M., Franz-Odendaal, T. A., Brasch, J., Castell, J. C. and Kaiser, T.** (2007). Tooth wear in captive giraffes (*Giraffa camelopardalis*): mesowear analysis classifies free-ranging specimens as browsers but captive ones as grazers. *Journal of Zoo and Wildlife Medicine* **38**, 433-45.

**Clavel, J., Merceron, G., Hristova, L., Spassov, N., Kovachev, D. and Escarguel, G.** (2012). On *Mesopithecus* habitat: Insights from late Miocene fossil vertebrate localities of Bulgaria. *Journal of Human Evolution* **63**, 162-179.

**Croft, D. A. and Weinstein, D.** (2008). The first application of the mesowear method to endemic South American ungulates (Notoungulata). *Palaeogeography, Palaeoclimatology, Palaeoecology* **269**, 103-114.

**Croitor, R. and Kaiser, T. M.** (2002). Functional morphology and diet preferences of fossil deer and paleolandscape reconstruction on early Pleistocene of Ceyssaguet. *Verhandlungen der Gesellschaft für Ökologie* **32**, 465.

**Curran, S. C. and Haile-Selassie, Y.** (2016). Paleoecological reconstruction of hominin-bearing middle Pliocene localities at Woranso-Mille, Ethiopia. *Journal of Human Evolution* **96**, 97-112.

**Danowitz, M., Hou, S., Mihlbachler, M., Hastings, V. and Solounias, N.** (2016). A combined-mesowear analysis of late Miocene giraffids from North Chinese and Greek localities of the Pikermian Biome. *Palaeogeography, Palaeoclimatology, Palaeoecology* **449**, 194-204.

**Daujeard, C., Vettese, D., Britton, K., Béarez, P., Boulbes, N., Crégut-Bonnoure, E., Desclaux, E., Lateur, N., Pike-Tay, A. and Rivals, F.** (2019). Neanderthal selective hunting of reindeer? The case study of Abri du Maras (south-eastern France). *Archaeological and Anthropological Sciences* **11**, 985-1011.

**DeMiguel, D.** (2016). Disentangling adaptive evolutionary radiations and the role of diet in promoting diversification on islands. *Scientific Reports* **6**, 29803.

**DeMiguel, D., Azanza, B. and Morales, J.** (2010). Trophic flexibility within the oldest *Cervidae* lineage to persist through the Miocene Climatic Optimum. *Palaeogeography, Palaeoclimatology, Palaeoecology* **289**, 81-92.

**DeMiguel, D., Azanza, B. and Morales, J.** (2011). Paleoenvironments and paleoclimate of the Middle Miocene of central Spain: A reconstruction from dental wear of ruminants. *Palaeogeography, Palaeoclimatology, Palaeoecology* **302**, 452-463.

**DeMiguel, D., Azanza, B. and Morales, J.** (2019). Regional impacts of global climate change: a local humid phase in central Iberia in a late Miocene drying world. *Palaeontology* **62**, 77-92.

**DeMiguel, D., Fortelius, M., Azanza, B. and Morales, J.** (2008). Ancestral feeding state of ruminants reconsidered: earliest grazing adaptation claims a mixed condition for *Cervidae*. *BMC Evolutionary Biology* **8**.

**DeMiguel, D., Quiralte, V., Azanza, B., Montoya, P. and Morales, J.** (2012). Dietary behaviour and competition for vegetal resources in two Early Miocene pecoran ruminants from Central Spain. *Geodiversitas* **34**, 425-444.

**DeMiguel, D. and Rook, L.** (2018). Understanding climate's influence on the extinction of *Oreopithecus* (late Miocene, Tusco-Sardinian paleobioprovince, Italy). *Journal of Human Evolution* **116**, 14-26.

**DeSantis, L. R. G., Alexander, J., Biedron, E. M., Johnson, P. S., Frank, A. S., Martin, J. M. and Williams, L.** (2018). Effects of climate on dental mesowear of extant koalas and two broadly distributed kangaroos throughout their geographic range. *PloS one* **13**, e0201962.

**Diana, P., Juha, S., Reinhard, Z. and Hervé, B.** (2020). Stable isotopic and mesowear reconstructions of paleodiet and habitat of the Middle and Late Pleistocene mammals in south-western Germany. *Quaternary Science Reviews* **227**, 106026.

**Díaz-Sibaja, R., Jiménez-Hidalgo, E., Ponce-Saavedra, J. and García-Zepeda, M. L.** (2018). A combined mesowear analysis of Mexican *Bison antiquus* shows a generalist diet with geographical variation. *Journal of Paleontology* **92**, 1130-1139.

**Domínguez-Rodrigo, M., Sánchez-Flores, A. J., Baquedano, E., Arriaza, M. M. C., Aramendi, J., Cobo, L., Organista, E. and Barba, R.** (2019). Constraining time and ecology on the Zinj paleolandscape: Microwear and mesowear analyses of the archaeofaunal remains of FLK Zinj and DS (Bed I), compared to FLK North (Bed I) and BK (Bed II) at Olduvai Gorge (Tanzania). *Quaternary International*.

**Dumouchel, L. and Bobe, R.** (2019). Paleoecological implications of dental mesowear and hypsodonty in fossil ungulates from Kanapoi. *Journal of Human Evolution* **Online**.

**Eronen, J. T., Evans, A. R., Fortelius, M. and Jernvall, J.** (2010). The impact of regional climate on the evolution of mammals: a case study using fossil horses. *Evolution: International Journal of Organic Evolution* **64**, 398-408.

**Eronen, J. T., Kaakinen, A., Liu, L.-P., Passey, B. H., Tang, H. and Zhang, Z.-Q.** (2014). Here be Dragons: Mesowear and tooth enamel isotopes of the classic Chinese “*Hipparion*” faunas from Baode, Shanxi Province, China. *Annales Zoologici Fennici* **51**, 227-456.

**Faith, J. T.** (2011). Late Quaternary dietary shifts of the Cape grysbok (*Raphicerus melanotis*) in southern Africa. *Quaternary Research* **75**, 159-165.

**Faith, J. T., Choiniere, J. N., Tryon, C. A., Peppe, D. J. and Fox, D. L.** (2011). Taxonomic status and paleoecology of *Rusingoryx atopocranion* (Mammalia, Artiodactyla), an extinct Pleistocene bovid from Rusinga Island, Kenya. *Quaternary Research* **75**, 697-707.

**Fortelius, M. and Solounias, N.** (2000). Functional characterization of ungulate molars using the abrasion-attrition wear gradient: a new method for reconstructing paleodiets. *American Museum Novitates* **3301**, 1-36.

**Franz-Odendaal, T. A. and Kaiser, T. M.** (2003). Differential mesowear in the maxillary and mandibular cheek dentition of some ruminants (Artiodactyla). *Annales Zoologici Fennici* **40**, 395-410.

**Franz-Odendaal, T. A., Kaiser, T. M. and Bernor, R. L.** (2003). Systematics and dietary evaluation of a fossil equid from South Africa. *South African Journal of Science* **99**, 453-459.

**Franz-Odendaal, T. A. and Solounias, N.** (2004). Comparative dietary evaluations of an extinct giraffid (*Sivatherium hendeyi*)(Mammalia, Giraffidae, Sivatheriinae) from Langebaanweg, South Africa (early Pliocene). *Geodiversitas* **26**, 675-685.

**Fraser, D. and Theodor, J. M.** (2010). The use of gross dental wear in dietary studies of extinct lagomorphs. *Journal of Paleontology* **84**, 720-729.

**Fraser, D. and Theodor, J. M.** (2013). Ungulate diets reveal patterns of grassland evolution in North America. *Palaeogeography, Palaeoclimatology, Palaeoecology* **369**, 409-421.

**Fraser, D., Zybutz, T., Lightner, E. and Theodor, J.** (2014). Ruminant mandibular tooth mesowear: A new scheme for increasing paleoecological sample sizes. *Journal of Zoology* **294**, 41-48.

**Gentry, A. W. and Kaiser, T. M.** (2009). The Bovidae of Dorn-Dürkheim 1, Germany (Turolian age). *Paläontologische Zeitschrift* **83**, 373-392.

**Hernesniemi, E., Blomstedt, K. and Fortelius, M.** (2011a). Multi-view stereo three-dimensional reconstruction of lower molars of Recent and Pleistocene rhinoceroses for mesowear analysis. *Palaeontologia Electronica* **14**, 1-15.

**Hernesniemi, E., Giaourtsakis, I. X., Evans, A. R. and Fortelius, M.** (2011b). Rhinocerotidae. In *Paleontology and Geology of Laetoli: Human Evolution in Context*, (ed. T. Harrison), pp. 275-294. Dordrecht: Springer.

**Hoffman, J. M.** (2006). Using stable carbon isotope, microwear, and mesowear analyses to determine the paleodiets of Neogene ungulates and the presence of c4 or c3 grasses in northern and central Florida, vol. PhD: University of Florida.

**Hulbert Jr, R. C., Czaplewski, N. J. and Webb, S. D.** (2005). New records of *Pseudhipparion simpsoni* (Mammalia, Equidae) from the late Hemphillian of Oklahoma and Florida. *Journal of Vertebrate Paleontology* **25**, 737-740.

**Jiménez-Hidalgo, E., Cabrera-Pérez, L., MacFadden, B. J. and Guerrero-Arenas, R.** (2013). First record of *Bison antiquus* from the Late Pleistocene of southern Mexico. *Journal of South American Earth Sciences* **42**, 83-90.

**Jiménez-Hidalgo, E., Carbot-Chanona, G., Guerrero-Arenas, R., Bravo-Cuevas, V. M., Holdridge, G. S. and Israde-Alcántara, I.** (2019). Species diversity and paleoecology of Late Pleistocene horses from southern Mexico. *Frontiers in Ecology and Evolution* **7**, 394.

**Joomun, S. C., Hooker, J. J. and Collinson, M. E.** (2008). Dental wear variation and implications for diet: an example from Eocene perissodactyls (Mammalia). *Palaeogeography, Palaeoclimatology, Palaeoecology* **263**, 92-106.

**Joomun, S. C., Hooker, J. J. and Collinson, M. E.** (2010). Changes in dental wear of *Plagiolophus minor* (Mammalia: Perissodactyla) across the Eocene–Oligocene transition. *Journal of Vertebrate Paleontology* **30**, 563-576.

**Jordana, X., Marín-Moratalla, N., DeMiguel, D., Kaiser, T. M. and Köhler, M.** (2012). Evidence of correlated evolution of hypsodonty and exceptional longevity in endemic insular mammals. *Proceedings of the Royal Society B* **279**, 3339-3346.

**Kahlke, R.-D. and Kaiser, T. M.** (2011). Generalism as a subsistence strategy: advantages and limitations of the highly flexible feeding traits of Pleistocene *Stephanorhinus hundsheimensis* (Rhinocerotidae, Mammalia). *Quaternary Science Reviews* **30**, 2250-2261.

**Kaiser, T. M.** (2003). The dietary regimes of two contemporaneous populations of *Hippotherium primigenium* (Perissodactyla, Equidae) from the Vallesian (Upper Miocene) of Southern Germany. *Palaeogeography, Palaeoclimatology, Palaeoecology* **198**, 381-402.

**Kaiser, T. M.** (2009). *Anchitherium aurelianense* (Equidae, Mammalia): a brachydont “dirty browser” in the community of herbivorous large mammals from Sandelzhausen (Miocene, Germany). *Paläontologische Zeitschrift* **83**, 131.

**Kaiser, T. M.** (2011). Feeding Ecology and Niche Partitioning of the Laetoli Ungulate Faunas. In *Paleontology and Geology of Laetoli: Human Evolution in Context: Volume 1: Geology, Geochronology, Paleoecology and Paleoenvironment*, (ed. T. Harrison), pp. 329-354. Dordrecht: Springer Netherlands.

**Kaiser, T. M. and Bernor, R. L.** (2006). The Baltavar *Hippotherium*: A mixed feeding Upper Miocene hipparion (Equidae, Perissodactyla) from Hungary (East-Central Europe). *Beiträge zur Paläontologie* **30**, 241-267.

**Kaiser, T. M., Bernor, R. L., Scott, R. S., Franzen, J. L. and Solounias, N.** (2003). New interpretations of the systematics and palaeoecology of the Dorn-Dürkheim 1 *Hipparions* (Late Miocene, Turolian Age [MN11]), Rheinhessen, Germany. *Senckenbergiana Lethaea* **83**, 103-133.

**Kaiser, T. M., Brasch, J., Castell, J. C., Schulz, E. and Clauss, M.** (2009). Tooth wear in captive wild ruminant species differs from that of free-ranging conspecifics. *Mammalian Biology - Zeitschrift für Säugetierkunde* **74**, 425-437.

**Kaiser, T. M., Clauss, M. and Schulz-Kornas, E.** (2015). A set of hypotheses on tribology of mammalian herbivore teeth. *Surface Topography: Metrology and Properties* **4**, 014003.

**Kaiser, T. M. and Croitor, R.** (2004). Ecological interpretations of early Pleistocene deer (Mammalia, Cervidae) from Ceyssaguet (Haute-Loire, France). *Geodiversitas* **26**, 661-674.

**Kaiser, T. M. and Fortelius, M.** (2003). Differential mesowear in occluding upper and lower molars: opening mesowear analysis for lower molars and premolars in hypsodont horses. *Journal of Morphology* **258**, 67-83.

**Kaiser, T. M. and Franz-Odendaal, T. A.** (2004). A mixed-feeding *Equus* species from the Middle Pleistocene of South Africa. *Quaternary Research* **62**, 316-323.

**Kaiser, T. M. and Kahlke, R.-D.** (2005). The highly flexible feeding strategy of *Stephanorhinus etruscus* (Falconer, 1859)(Rhinocerotidae, Mammalia) during the early Middle Pleistocene in Central Europe. *Berichte des Institutes für Erdwissenschaften, Karl-Franzens-Universität Graz* **10**, 50-53.

**Kaiser, T. M., Müller, D. W. H., Fortelius, M., Schulz, E., Codron, D. and Clauss, M.** (2013). Hypsodonty and tooth facet development in relation to diet and habitat in herbivorous ungulates: implications for understanding tooth wear. *Mammal Review* **43**, 34-46.

**Kaiser, T. M. and Rössner, G. E.** (2007). Dietary resource partitioning in ruminant communities of Miocene wetland and karst palaeoenvironments in Southern Germany. *Palaeogeography, Palaeoclimatology, Palaeoecology* **252**, 424-439.

**Kaiser, T. M. and Schulz, E.** (2006). Tooth wear gradients in zebras as an environmental proxy—a pilot study. *Mitteilungen aus dem Hamburgischen Zoologischen Museum und Institut* **103**, 187-210.

**Kaiser, T. M. and Solounias, N.** (2003). Extending the tooth mesowear method to extinct and extant equids. *Geodiversitas* **25**, 321-345.

**Kaiser, T. M., Solounias, N., Fortelius, M., Bernor, R. L. and Schrenk, F.** (2000). Tooth mesowear analysis on *Hippotherium primigenium* from the Vallesian Dinotheriensande (Germany). *Carolinea: Beiträge zur naturkundlichen Forschung in Südwestdeutschland* **58**, 103-114.

**Kaiser, T. M., Uerpmann, H.-P. and Schulz-Kornas, E.** (2008). The Diet of Neolithic Wild Asses (*Equus africanus,* Equinae, Perissodactyla) from BHS 18 (Sharjah, United Arab Emirates). In *The Natural Environment of Jebel al-Buhais: Past and Present*, eds. H.-P. Uerpmann M. Uerpmann and S. Abboud Jasim), pp. 133-142: Institut für Ur- und Frühgeschichte und Archäologie des Mittelalters Universität Tübingen Germany and Kerns Verlag Tübingen.

**Koufos, G. D.** (2009). The Neogene cercopithecids (Mammalia, Primates) of Greece. *Geodiversitas* **31**, 817-851.

**Kropacheva, Y. E., Sibiryakov, P. A., Smirnov, N. G. and Zykov, S. V.** (2017). Variants of tooth mesowear in *Microtus* voles as indicators of food hardness and abrasiveness. *Russian Journal of Ecology* **48**, 73-80.

**Kubo, M. O. and Yamada, E.** (2014). The inter-relationship between dietary and environmental properties and tooth wear: comparisons of mesowear, molar wear rate, and hypsodonty index of extant sika deer populations. *Plos One* **9**, e90745.

**Kubo, M. O., Yamada, E., Fujita, M. and Oshiro, I.** (2015). Paleoecological reconstruction of Late Pleistocene deer from the Ryukyu Islands, Japan: Combined evidence of mesowear and stable isotope analyses. *Palaeogeography, Palaeoclimatology, Palaeoecology* **435**, 159-166.

**Loffredo, L. F. and DeSantis, L. R.** (2014). Cautionary lessons from assessing dental mesowear observer variability and integrating paleoecological proxies of an extreme generalist *Cormohipparion emsliei*. *Palaeogeography, Palaeoclimatology, Palaeoecology* **395**, 42-52.

**López-García, J. M., Blain, H.-A., Burjachs, F., Ballesteros, A., Allué, E., Cuevas-Ruiz, G. E., Rivals, F., Blasco, R., Morales, J. I. and Hidalgo, A. R.** (2012). A multidisciplinary approach to reconstructing the chronology and environment of southwestern European Neanderthals: the contribution of Teixoneres cave (Moià, Barcelona, Spain). *Quaternary Science Reviews* **43**, 33-44.

**Louys, J., Ditchfield, P., Meloro, C., Elton, S. and Bishop, L. C.** (2012). Stable isotopes provide independent support for the use of mesowear variables for inferring diets in African antelopes. *Proceedings of the Royal Society of London B: Biological Sciences* **279**, 4441-4446.

**Louys, J., Meloro, C., Elton, S., Ditchfield, P. and Bishop, L. C.** (2011). Mesowear as a means of determining diets in African antelopes. *Journal of Archaeological Science* **38**, 1485-1495.

**MacFadden, B. J.** (2009). Three-toed browsing horse *Anchitherium* (Equidae) from the Miocene of Panama. *Journal of Paleontology* **83**, 489-492.

**Maniakas, I. and Kostopoulos, D. S.** (2017). Morphometric-palaeoecological discrimination between Bison populations of the western Palaearctic. *Geobios* **50**, 155-171.

**Marder, O., Yeshurun, R., Lupu, R., Bar‐Oz, G., Belmaker, M., Porat, N., Ron, H. and Frumkin, A.** (2011). Mammal remains at Rantis Cave, Israel, and Middle–Late Pleistocene human subsistence and ecology in the Southern Levant. *Journal of Quaternary Science* **26**, 769-780.

**Marín-Leyva, A. H., DeMiguel, D., García-Zepeda, M. L., Ponce-Saavedra, J., Arroyo-Cabrales, J., Schaaf, P. and Alberdi, M. T.** (2016). Dietary adaptability of Late Pleistocene *Equus* from west central Mexico. *Palaeogeography, Palaeoclimatology, Palaeoecology* **441**, 748-757.

**Marom, N., Garfinkel, Y. and Bar-Oz, G.** (2018). Times in between: A zooarchaeological analysis of ritual in Neolithic Sha'ar Hagolan. *Quaternary International* **464**, 216-225.

**Martínez-Polanco, M. F., Rivals, F. and Cooke, R. G.** (2019). Behind white-tailed deer teeth: A micro-and mesowear analysis from three Panamanian pre-Columbian archaeological sites. *Quaternary International*.

**Martínez‐Pérez, C., Rayfield, E. J., Purnell, M. A. and Donoghue, P. C.** (2014). Finite element, occlusal, microwear and microstructural analyses indicate that conodont microstructure is adapted to dental function. *Palaeontology* **57**, 1059-1066.

**McLennan, L.** (2018). Tooth Wear, Microwear and Diet in Elasmobranchs, vol. PhD. University of Leicester: Department of Geology.

**Meachen, J. A.** (2003). A new species of *Hemiauchenia* (Camelidae; Lamini) from the Plio-Pleistocene of Florida. In *Zoology*, vol. MSc: University of Florida.

**Melcher, M., Wolf, D. and Bernor, R. L.** (2013). The evolution and paleodiet of the *Eurygnathohippus feibeli* lineage in Africa. *Paläontologische Zeitschrift* **88**, 99-110.

**Mendes-Oliveira, A. C., de Maria, S. L., Soares de Lima, R. C., Fernandes, A. S., de Almeida, P. C. and Montag, L. F.** (2012). Testing simple criteria for age estimation of six hunted mammal species in the Brazilian Amazon. *Mastozoología neotropical* **19**, 105-116.

**Merceron, G.** (2009). The early Vallesian vertebrates of Atzelsdorf (Late Miocene, Austria). 13. Dental wear patterns of herbivorous ungulates as ecological indicators. *Annalen des Naturhistorischen Museums in Wien* **111**, 647-660.

**Merceron, G., Escarguel, G., Angibault, J.-M. and Verheyden-Tixier, H.** (2010). Can dental microwear textures record inter-individual dietary variations? *PLoS One* **5**, e9542.

**Merceron, G., Schulz, E., Kordos, L. and Kaiser, T. M.** (2007). Paleoenvironment of *Dryopithecus brancoi* at Rudabánya, Hungary: evidence from dental meso-and micro-wear analyses of large vegetarian mammals. *Journal of Human Evolution* **53**, 331-349.

**Mihlbachler, M. C., Campbell, D., Chen, C., Ayoub, M. and Kaur, P.** (2018). Microwear–mesowear congruence and mortality bias in rhinoceros mass-death assemblages. *Paleobiology* **44**, 131-154.

**Mihlbachler, M. C., Rivals, F., Solounias, N. and Semprebon, G. M.** (2011). Dietary change and evolution of horses in North America. *Science* **331**, 1178-1181.

**Mihlbachler, M. C. and Solounias, N.** (2006). Coevolution of tooth crown height and diet in oreodonts (Merycoidodontidae, Artiodactyla) examined with phylogenetically independent contrasts. *Journal of Mammalian Evolution* **13**, 11-36.

**Nakaya, H., Takai, M., Fukuchi, A. and Ogino, S.** (2009). A preliminary report on some fossil mammals (Equidae, Perissodactyla and Hyracoidea) from the Pliocene Udunga fauna, Transbaikalia, Russia. *Asian Paleoprimatology* **5**, 99-104.

**Palombo, M. R.** (2005). Food habits of *"Praemegaceros" cazioti* (Depéret, 1897) from Dragonara Cave (NW Sardinia, Italy) inferred from cranial morphology and dental wear. In *Proceedings of the International Symposium" Insular Vertebrate Evolution: the Palaeontological Approach": September, 16-19 Mallorca*, pp. 233-245: Societat d'Història Natural de les Balears.

**Pérez-Crespo, V. A., Barrón-Ortiz, C. R., Arroyo-Cabrales, J., Morales-Puente, P., Cienfuegos-Alvarado, E. and Otero, F. J.** (2016). Preliminary data on the diet and habitat preferences of *Capromeryx mexicana* (Mammalia: Antilocapridae) from the late Pleistocene of Cedral, San Luis Potosí, Mexico. *The Southwestern Naturalist* **61**, 152-156.

**Pokines, J. T., Lister, A. M., Ames, C. J., Nowell, A. and Cordova, C. E.** (2018). Faunal remains from recent excavations at Shishan Marsh 1 (SM1), a Late Lower Paleolithic open-air site in the Azraq Basin, Jordan. *Quaternary Research* **91**, 768-791.

**Purnell, M. A. and Jones, D.** (2012). Quantitative analysis of conodont tooth wear and damage as a test of ecological and functional hypotheses. *Paleobiology* **38**, 605-626.

**Pushkina, D., Saarinen, J., Ziegler, R. and Bocherens, H.** (2020). Stable isotopic and mesowear reconstructions of paleodiet and habitat of the Middle and Late Pleistocene mammals in south-western Germany. *Quaternary Science Reviews* **227**, 106026.

**Renaud, A. and Rivals, F.** (2007). Un petit boeuf médiéval identifié dans un silo. *Archéologie du Midi Médiéval* **25**, 181-187.

**Rivals, F.** (2012). Ungulate feeding ecology and middle Pleistocene paleoenvironments at Hundsheim and Deutsch-Altenburg 1 (eastern Austria). *Palaeogeography, Palaeoclimatology, Palaeoecology* **317**, 27-31.

**Rivals, F. and Álvarez-Lao, D. J.** (2018). Ungulate dietary traits and plasticity in zones of ecological transition inferred from late Pleistocene assemblages at Jou Puerta and Rexidora in the Cantabrian Region of northern Spain. *Palaeogeography, Palaeoclimatology, Palaeoecology* **499**, 123-130.

**Rivals, F. and Athanassiou, A.** (2008). Dietary adaptations in an ungulate community from the late Pliocene of Greece. *Palaeogeography, Palaeoclimatology, Palaeoecology* **265**, 134-139.

**Rivals, F., Gardeisen, A. and Cantuel, J.** (2011a). Domestic and wild ungulate dietary traits at Kouphovouno (Sparta, Greece): implications for livestock management and paleoenvironment in the Neolithic. *Journal of Archaeological Science* **38**, 528-537.

**Rivals, F., Julien, M.-A., Kuitems, M., Van Kolfschoten, T., Serangeli, J., Drucker, D. G., Bocherens, H. and Conard, N. J.** (2015). Investigation of equid paleodiet from Schöningen 13 II-4 through dental wear and isotopic analyses: Archaeological implications. *Journal of Human Evolution* **89**, 129-137.

**Rivals, F., Kitagawa, K., Julien, M.-A., Patou-Mathis, M., Bessudnov, A. A. and Bessudnov, A. N.** (2018a). Straight from the horse's mouth: High-resolution proxies for the study of horse diet and its relation to the seasonal occupation patterns at Divnogor'ye 9 (Middle Don, Central Russia). *Quaternary International* **474**, 146-155.

**Rivals, F. and Lister, A. M.** (2016). Dietary flexibility and niche partitioning of large herbivores through the Pleistocene of Britain. *Quaternary Science Reviews* **146**, 116-133.

**Rivals, F., Mihlbachler, M. C. and Solounias, N.** (2007a). Effect of ontogenetic-age distribution in fossil and modern samples on the interpretation of ungulate paleodiets using the mesowear method. *Journal of Vertebrate Paleontology* **27**, 763-767.

**Rivals, F., Mihlbachler, M. C., Solounias, N., Mol, D., Semprebon, G. M., de Vos, J. and Kalthoff, D. C.** (2010). Palaeoecology of the Mammoth Steppe fauna from the late Pleistocene of the North Sea and Alaska: Separating species preferences from geographic influence in paleoecological dental wear analysis. *Palaeogeography, Palaeoclimatology, Palaeoecology* **286**, 42-54.

**Rivals, F., Moncel, M.-H. and Patou-Mathis, M.** (2009a). Seasonality and intra-site variation of Neanderthal occupations in the Middle Palaeolithic locality of Payre (Ardèche, France) using dental wear analyses. *Journal of Archaeological Science* **36**, 1070-1078.

**Rivals, F., Rindel, D. and Belardi, J. B.** (2013). Dietary ecology of extant guanaco (*Lama guanicoe*) from Southern Patagonia: seasonal leaf browsing and its archaeological implications. *Journal of Archaeological Science* **40**, 2971-2980.

**Rivals, F., Sanz, M. and Daura, J.** (2016). First reconstruction of the dietary traits of the Mediterranean deer (*Haploidoceros mediterraneus*) from the Cova del Rinoceront (NE Iberian Peninsula). *Palaeogeography, Palaeoclimatology, Palaeoecology* **449**, 101-107.

**Rivals, F., Schulz, E. and Kaiser, T. M.** (2008). Climate-related dietary diversity of the ungulate faunas from the middle Pleistocene succession (OIS 14-12) at the Caune de l'Arago (France). *Paleobiology* **34**, 117-127.

**Rivals, F., Schulz, E. and Kaiser, T. M.** (2009b). Late and middle Pleistocene ungulates dietary diversity in Western Europe indicate variations of Neanderthal paleoenvironments through time and space. *Quaternary Science Reviews* **28**, 3388-3400.

**Rivals, F., Schulz, E. and Kaiser, T. M.** (2009c). A new application of dental wear analyses: estimation of duration of hominid occupations in archaeological localities. *Journal of Human Evolution* **56**, 329-339.

**Rivals, F. and Semprebon, G. M.** (2006). A comparison of the dietary habits of a large sample of the Pleistocene pronghorn *Stockoceros onusrosagris* from the Papago Springs Cave in Arizona to the modern *Antilocapra americana*. *Journal of Vertebrate Paleontology* **26**, 495-500.

**Rivals, F. and Semprebon, G. M.** (2010). What can incisor microwear reveal about the diet of ungulates? *Mammalia* **74**, 401-406.

**Rivals, F. and Semprebon, G. M.** (2012). Paleoindian subsistence strategies and late Pleistocene paleoenvironments in the northeastern and southwestern United States: a tooth wear analysis. *Journal of Archaeological Science* **39**, 1608-1617.

**Rivals, F. and Semprebon, G. M.** (2017). Latitude matters: an examination of behavioural plasticity in dietary traits amongst extant and Pleistocene *Rangifer tarandus*. *Boreas* **46**, 254-263.

**Rivals, F., Solounias, N. and Mihlbachler, M. C.** (2007b). Evidence for geographic variation in the diets of late Pleistocene and early Holocene Bison in North America, and differences from the diets of recent *Bison*. *Quaternary Research* **68**, 338-346.

**Rivals, F., Solounias, N. and Schaller, G. B.** (2011b). Diet of Mongolian gazelles and Tibetan antelopes from steppe habitats using premaxillary shape, tooth mesowear and microwear analyses. *Mammalian Biology-Zeitschrift für Säugetierkunde* **76**, 358-364.

**Rivals, F. and Takatsuki, S.** (2015). Within-island local variations in tooth wear of sika deer (*Cervus nippon centralis*) in northern Japan. *Mammalian Biology* **80**, 333-339.

**Rivals, F., Takatsuki, S., Albert, R. M. and Macià, L.** (2014). Bamboo feeding and tooth wear of three sika deer (*Cervus nippon*) populations from northern Japan. *Journal of Mammalogy* **95**, 1043-1053.

**Rivals, F., Uno, K. T., Bibi, F., Pante, M. C., Njau, J. and de la Torre, I.** (2018b). Dietary traits of the ungulates from the HWK EE site at Olduvai Gorge (Tanzania): Diachronic changes and seasonality. *Journal of Human Evolution* **120**, 203-214.

**Rivals, F., Uzunidis, A., Sanz, M. and Daura, J.** (2017). Faunal dietary response to the Heinrich Event 4 in southwestern Europe. *Palaeogeography, Palaeoclimatology, Palaeoecology* **473**, 123-130.

**Rivals, F. and Ziegler, R.** (2018). High-resolution paleoenvironmental context for human occupations during the Middle Pleistocene in Europe (MIS 11, Germany). *Quaternary Science Reviews* **188**, 136-142.

**Rowan, J., Faith, J. T., Gebru, Y. and Fleagle, J. G.** (2015). Taxonomy and paleoecology of fossil *Bovidae* (Mammalia, Artiodactyla) from the Kibish Formation, southern Ethiopia: Implications for dietary change, biogeography, and the structure of the living bovid faunas of East Africa. *Palaeogeography, Palaeoclimatology, Palaeoecology* **420**, 210-222.

**Rozzi, R., Winkler, D. E., De Vos, J., Schulz, E. and Palombo, M. R.** (2013). The enigmatic bovid *Duboisia santeng* (Dubois, 1891) from the Early–Middle Pleistocene of Java: A multiproxy approach to its paleoecology. *Palaeogeography, Palaeoclimatology, Palaeoecology* **377**, 73-85.

**Saarinen, J., Eronen, J., Fortelius, M., Seppä, H. and Lister, A. M.** (2016). Patterns of diet and body mass of large ungulates from the Pleistocene of Western Europe, and their relation to vegetation. *Palaeontologia Electronica* **19**, 1-58.

**Saarinen, J. and Karme, A.** (2017). Tooth wear and diets of extant and fossil xenarthrans (Mammalia, Xenarthra) – Applying a new mesowear approach. *Palaeogeography, Palaeoclimatology, Palaeoecology* **476**, 42-54.

**Saarinen, J., Karme, A., Cerling, T., Uno, K., Säilä, L., Kasiki, S., Ngene, S., Obari, T., Mbua, E. and Manthi, F. K.** (2015). A new tooth wear–based dietary analysis method for proboscidea (Mammalia). *Journal of Vertebrate Paleontology* **35**, e918546.

**Saarinen, J. and Lister, A. M.** (2016). Dental mesowear reflects local vegetation and niche separation in Pleistocene proboscideans from Britain. *Journal of Quaternary Science* **31**, 799-808.

**Sánchez-Hernández, C., Rivals, F., Blasco, R. and Rosell, J.** (2016). Tale of two timescales: combining tooth wear methods with different temporal resolutions to detect seasonality of Palaeolithic hominin occupational patterns. *Journal of Archaeological Science: Reports* **6**, 790-797.

**Sanz, M., Rivals, F., García, D. and Zilhão, J.** (2019). Hunting strategy and seasonality in the last interglacial occupation of Cueva Antón (Murcia, Spain). *Archaeological and Anthropological Sciences* **Online**, 1-18.

**Schubert, B. W.** (2007). Dental mesowear and the palaeodiets of bovids from Makapansgat Limeworks Cave, South Africa. *Palaentologia Africana* **43**, 43-50.

**Schulz, E. and Fahlke, J. M.** (2009). The diet of *Metaschizotherium bavaricum* (Chalicotheriidae, Mammalia) from the MN 5 of Sandelzhausen (Germany) implied by the mesowear method. *Palaeontologische Zeitschrift* **83**, 175-181.

**Schulz, E., Fahlke, J. M., Merceron, G. and Kaiser, T. M.** (2007a). Feeding ecology of the *Chalicotheriida*e (Mammalia, Perissodactyla, Ancylopoda). Results from dental micro-and mesowear analyses. *Verhandlungen des Naturwissenschaftlichen Vereins zu Hamburg* **43**, 5-31.

**Schulz, E., Fraas, S., Kaiser, T. M., Cunningham, P. L., Ismail, K. and Wronski, T.** (2013). Food preferences and tooth wear in the sand gazelle (*Gazella marica*). *Mammalian Biology-Zeitschrift für Säugetierkunde* **78**, 55-62.

**Schulz, E., Kaiser, T., Stubbe, A., Stubbe, M., Samjaa, R., Batsaikhan, N. and Wussow, J.** (2007b). Comparative demography and dietary resource partitioning of two wild ranging Asiatic equid populations. *Erforschung biologischer Ressourcen der Mongolei* **10**, 77-90.

**Schulz, E. and Kaiser, T. M.** (2007). Feeding strategy of the Urus *Bos primigenius Bojanus*, 1827 from the Holocene of Denmark. *Courier forschungsinstitut Senckenberg* **259**, 155.

**Schulz, E. and Kaiser, T. M.** (2013). Historical distribution, habitat requirements and feeding ecology of the genus *Equus* (Perissodactyla). *Mammal Review* **43**, 111-123.

**Sealy, J., Naidoo, N., Hare, V. J., Brunton, S. and Faith, J. T.** (2019). Climate and ecology of the palaeo-Agulhas Plain from stable carbon and oxygen isotopes in bovid tooth enamel from Nelson Bay Cave, South Africa. *Quaternary Science Reviews*, 105974.

**Semprebon, G., Janis, C. and Solounias, N.** (2004). The diets of the *Dromomerycidae* (Mammalia: Artiodactyla) and their response to Miocene vegetational change. *Journal of Vertebrate Paleontology* **24**, 427-444.

**Semprebon, G. M. and Rivals, F.** (2007). Was grass more prevalent in the pronghorn past? An assessment of the dietary adaptations of Miocene to recent *Antilocapridae* (Mammalia: Artiodactyla). *Palaeogeography, Palaeoclimatology, Palaeoecology* **253**, 332-347.

**Semprebon, G. M. and Rivals, F.** (2010). Trends in the paleodietary habits of fossil camels from the Tertiary and Quaternary of North America. *Palaeogeography, Palaeoclimatology, Palaeoecology* **295**, 131-145.

**Sewell, L., Merceron, G., Hopley, P., Zipfel, B. and Reynolds, S. C.** (2019). Using springbok (*Antidorcas*) dietary proxies to reconstruct inferred palaeovegetational changes over 2 million years in Southern Africa. *Journal of Archaeological Science: Reports* **23**, 1014-1028.

**Seyler, H. C.** (2018). Comparing Dietary Differences Among the Hornless Artiodactyl Ruminants *Hypertragulus*, *Hypisodus*, and *Leptomeryx* from the Early Oligocene (Orellan) Brule Formation, Toadstool Geologic Park, Nebraska, vol. MSc: University of Colorado at Boulder.

**Solounias, N. and Danowitz, M.** (2016). The Giraffidae of Maragheh and the identification of a new species of Honanotherium. *Palaeobiodiversity and Palaeoenvironments* **96**, 489-506.

**Solounias, N., Semprebon, G., Mihlbachler, M. and Rivals, F.** (2013). Paleodietary comparisons of ungulates between the late Miocene of China, and Pikermi and Samos in Greece. In *Fossil Mammals of Asia: Neogene Biostratigraphy and Chronology.* , eds. X. Wang L. J. Flynn and M. Fortelius), pp. 676-692. New York: Columbia University Press.

**Solounias, N., Tariq, M., Hou, S., Danowitz, M. and Harrison, M.** (2014). A new method of tooth mesowear and a test of it on domestic goats. *Annales Zoologici Fennici* **51**, 111-118.

**Stauffer, J. B., Clauss, M., Müller, D. W. H., Hatt, J.-M. and Ackermans, N. L.** (2019). Testing mesowear III on experimentally fed goats (*Capra aegagrus hircus*). *Annales Zoologici Fennici* **56**, 85-91.

**Stimpson, C. M., Lister, A., Parton, A., Clark-Balzan, L., Breeze, P. S., Drake, N. A., Groucutt, H. S., Jennings, R., Scerri, E. M. and White, T. S.** (2016). Middle Pleistocene vertebrate fossils from the Nefud Desert, Saudi Arabia: implications for biogeography and palaeoecology. *Quaternary Science Reviews* **143**, 13-36.

**Strani, F., DeMiguel, D., Alba, D. M., Moyà-Solà, S., Bellucci, L., Sardella, R. and Madurell-Malapeira, J.** (2019a). The effects of the “0.9 Ma event” on the Mediterranean ecosystems during the Early-Middle Pleistocene transition as revealed by dental wear patterns of fossil ungulates. *Quaternary Science Reviews* **210**, 80-89.

**Strani, F., DeMiguel, D., Bona, F., Sardella, R., Biddittu, I., Bruni, L., De Castro, A., Guadagnoli, F. and Bellucci, L.** (2018a). Ungulate dietary adaptations and palaeoecology of the Middle Pleistocene site of Fontana Ranuccio (Anagni, Central Italy). *Palaeogeography, Palaeoclimatology, Palaeoecology* **496**, 238-247.

**Strani, F., DeMiguel, D., Sardella, R. and Bellucci, L.** (2015). Paleoenvironments and climatic changes in the Italian Peninsula during the Early Pleistocene: evidence from dental wear patterns of the ungulate community of Coste San Giacomo. *Quaternary Science Reviews* **121**, 28-35.

**Strani, F., DeMiguel, D., Sardella, R. and Bellucci, L.** (2018b). Resource and niche differentiation mechanisms by sympatric Early Pleistocene ungulates: the case study of Coste San Giacomo. *Quaternary International* **481**, 157-163.

**Strani, F., Pushkina, D., Bocherens, H., Bellucci, L., Sardella, R. and DeMiguel, D.** (2019b). Dietary adaptations of Early and Middle Pleistocene equids from the Anagni basin (Frosinone, central Italy). *Frontiers in Ecology and Evolution* **7**, 176.

**Stynder, D. D.** (2009). The diets of ungulates from the hominid fossil-bearing site of Elandsfontein, Western Cape, South Africa. *Quaternary Research* **71**, 62-70.

**Stynder, D. D.** (2011). Fossil bovid diets indicate a scarcity of grass in the Langebaanweg E Quarry (South Africa) late Miocene/early Pliocene environment. *Paleobiology* **37**, 126-139.

**Suttner, T. J., Kido, E. and Briguglio, A.** (2017). A new icriodontid conodont cluster with specific mesowear supports an alternative apparatus motion model for Icriodontidae. *Journal of Systematic Palaeontology* **16**, 909-926.

**Tariq, M. and Jahan, N.** (2014). Dietary evaluations and paleoecology of an extinct Giraffid (*Giraffokeryx punjabiensis*) from Siwaliks of Pakistan. *Journal of Animal and Plant Sciences* **24**, 1355-1365.

**Taylor, L. A., Kaiser, T. M., Schwitzer, C., Müller, D. W. H., Codron, D., Clauss, M. and Schulz, E.** (2013). Detecting inter-cusp and inter-tooth wear patterns in Rhinocerotids. *PLoS One* **8**, e80921.

**Taylor, L. A., Müller, D. W. H., Schwitzer, C., Kaiser, T. M., Castell, J. C., Clauss, M. and Schulz-Kornas, E.** (2016). Comparative analyses of tooth wear in free‐ranging and captive wild equids. *Equine Veterinary Journal* **48**, 240-245.

**Taylor, L. A., Müller, D. W. H., Schwitzer, C., Kaiser, T. M., Codron, D., Schulz, E. and Clauss, M.** (2014). Tooth wear in captive rhinoceroses (*Diceros, Rhinoceros, Ceratotherium*: Perissodactyla) differs from that of free-ranging conspecics. *Contributions to Zoology* **83**, 107-117.

**Tütken, T., Kaiser, T. M., Vennemann, T. and Merceron, G.** (2013). Opportunistic feeding strategy for the earliest Old World hypsodont equids: evidence from stable isotope and dental wear proxies. *PLoS One* **8**, e74463.

**Ulbricht, A., Maul, L. C. and Schulz, E.** (2015). Can mesowear analysis be applied to small mammals? A pilot-study on leporines and murines. *Mammalian Biology-Zeitschrift für Säugetierkunde* **80**, 14-20.

**Uno, K. T., Rivals, F., Bibi, F., Pante, M., Njau, J. and de la Torre, I.** (2018). Large mammal diets and paleoecology across the Oldowan–Acheulean transition at Olduvai Gorge, Tanzania from stable isotope and tooth wear analyses. *Journal of Human Evolution* **120**, 76-91.

**Uzunidis, A., Rivals, F. and Brugal, J.-P.** (2017). Relation between morphology and dietary traits in horse jugal upper teeth during the Middle Pleistocene in Southern France. *Quaternaire* **28**, 303-312.

**Valli, A. M. and Palombo, M. R.** (2005). Le régime alimentaire du Cervidae (Mammalia) *Eucladoceros ctenoides* (Nesti 1841) reconstitué par la morphologie du crâne et par l’usure dentaire. *Eclogae Geologicae Helvetiae* **98**, 133-143.

**Valli, A. M. and Palombo, M. R.** (2008). Feeding behaviour of middle-size deer from the Upper Pliocene site of Saint-Vallier (France) inferred by morphological and micro/mesowear analysis. *Palaeogeography, Palaeoclimatology, Palaeoecology* **257**, 106-122.

**van Asperen, E. N. and Kahlke, R.-D.** (2015). Dietary variation and overlap in Central and Northwest European *Stephanorhinus kirchbergensis* and *S. hemitoechus* (Rhinocerotidae, Mammalia) influenced by habitat diversity:“You'll have to take pot luck!”(proverb). *Quaternary Science Reviews* **107**, 47-61.

**van Asperen, E. N. and Kahlke, R.-D.** (2017). Dietary traits of the late early Pleistocene *Bison menneri* (Bovidae, Mammalia) from its type site Untermassfeld (Central Germany) and the problem of Pleistocene ‘wood bison’. *Quaternary Science Reviews* **177**, 299-313.

**Viranta, S. and Mannermaa, K.** (2014). Mesowear analysis on Finnish medieval horses. *Annales Zoologici Fennici* **51**, 119-123.

**White, T. D., Ambrose, S. H., Suwa, G., Su, D. F., DeGusta, D., Bernor, R. L., Boisserie, J.-R., Brunet, M., Delson, E. and Frost, S.** (2009). Macrovertebrate paleontology and the Pliocene habitat of *Ardipithecus ramidus*. *Science* **326**, 67-93.

**Widga, C.** (2006). Niche variability in late Holocene bison: a perspective from Big Bone Lick, KY. *Journal of Archaeological Science* **33**, 1237-1255.

**Winkler, D. E. and Kaiser, T. M.** (2011). A case study of seasonal, sexual and ontogenetic divergence in the feeding behaviour of the moose (*Alces alces,* Linné, 1758). *Verhandlungen des Naturwissenschaftlichen Vereins Hamburg* **46**, 331-348.

**Wirkner, M. and Hertler, C.** (2019). Feeding ecology of Late Pleistocene *Muntiacus muntjak* in the Padang Highlands (Sumatra). *Comptes Rendus Palevol* **Online**.

**Wolf, D., Nelson, S. V., Schwartz, H. L., Semprebon, G. M., Kaiser, T. M. and Bernor, R. L.** (2010). Taxonomy and paleoecology of the Pleistocene Equidae from Makuyuni, northern Tanzania. *Palaeodiversity* **3**, 249-269.

**Wolf, D., Semprebon, G. M. and Bernor, R. L.** (2012). New observations on the paleodiet of the late Miocene Höwenegg (Hegau, Germany) *Hippotherium primigenium* (Mammalia, Equidae). *Bollettino della Società Paleontologica Italiana* **51**, 186.

**Wronski, T. and Schulz-Kornas, E.** (2015). The Farasan gazelle—A frugivorous browser in an arid environment? *Mammalian Biology* **80**, 87-95.

**Yamada, E.** (2013). Effects of dietary differences between sympatric Japanese serow and sika deer on environmental reconstruction by means of mesowear analysis. *Annales Zoologici Fennici* **50**, 200-209.

**Yamada, E., Hasumi, E., Miyazato, N., Akahoshi, M., Watabe, M. and Nakaya, H.** (2016). Mesowear analyses of sympatric ungulates from the late Miocene Maragheh, Iran. *Palaeobiodiversity and Palaeoenvironments* **96**, 445-452.
